# Escitalopram in patients with psoriasis

**DOI:** 10.1192/j.eurpsy.2021.650

**Published:** 2021-08-13

**Authors:** M. Artemieva, I. Danilin, Z. Ziewozinska, R. Suleimanov, A. Lazukova

**Affiliations:** 1 Psychiatry And Medical Psychology, RUDN University, Moscow, Russian Federation; 2 Dermatology And Venereology, Pirogov Russian National Research Medical University, Moscow, Russian Federation

**Keywords:** Escitalopram, psoriasis, psychosomatic disease

## Abstract

**Introduction:**

Psoriasis is a chronic skin disease, affecting up to 2-4% of population. The majority of investigations agree that this disease appears to be a result of confluence of genetic, allergenic and emotional factors. There is usually more than one trigger that leads to the manifestation or exacerbation of symptoms. Psychogenic factors are clearly in a pattern here. Some personality traits may lead to instability of the emotional sphere that can act alternately as a cause, then as an aftereffect of exacerbation of the chronic process, forming a so called “vicious circle” of psychosomatic disease.

**Objectives:**

To discover the impact of escitalopram on the symptoms of psoriasis.

**Methods:**

14 patients were diagnosed with psoriasis. All patients underwent psychiatric interviewing and psychological testing (STAI and HADS). The results revealed increased trait anxiety levels among 8 of them with moderate severity of the process according to SCORAD index. 3 cases demonstrated symptoms of clinically expressed and 4 subclinical levels of depression. Escitalopram (10 to 20 mg. daily) was administered along with common dermatologic therapy in these patients.

**Results:**

Patients treated with escitalopram showed a reduction of anxiety and depression tests scores as well as the values some dermatological symptoms such as pruritus.

**Conclusions:**

These preliminary results of 3 months study gives us a hope of successful psychopharmacological treatment of psoriasis in cooperation with dermatologists. Finding intercommunications in pathogenesis of skin and emotional disorders could optimize the treatment and improve patient’s quality of life. The publication was prepared with the support of the “RUDN University Program 5-100”.

